# Prescription drug dependence with and without concurrent illicit drug use: a multicenter cross-sectional survey among an addiction treatment seeking population

**DOI:** 10.3389/fpsyt.2023.1133606

**Published:** 2023-06-01

**Authors:** Asma Nawaz, Suzanne Nielsen, Tahir Mehmood, Abdullah Abdullah, Ali Ahmed, Waseem Ullah, Ahmad Khan

**Affiliations:** ^1^Department of Pharmacy, Quaid-i-Azam University, Islamabad, Pakistan; ^2^Monash Addiction Research Centre, Eastern Health Clinical School, Monash University, Melbourne, VIC, Australia; ^3^School of Natural Sciences, National University of Science and Technology, Islamabad, Pakistan; ^4^Drug Regulatory Authority of Pakistan, Islamabad, Pakistan; ^5^School of Pharmacy, Monash University, Bandar Sunway, Selangor, Malaysia; ^6^Department of Pharmacy Practice, Shifa College of Pharmaceutical Sciences, Shifa Tameer-e-Millat University, Islamabad, Pakistan

**Keywords:** prescription drug use, illicit drug use, benzodiazepines, opioids, narcotics analgesics

## Abstract

**Background:**

Dependence on prescription drugs and illicit drugs imposes a global health and social burden. Despite accumulating evidence of prescription drugs and illicit drugs dependence, none of the systematized studies has explored the magnitude of this problem in Pakistan. The aim is to investigate the extent and associated factors of prescription drug dependence (PDD), as opposed to concomitant prescription drug dependence and illicit drug use (PIDU), within a sample of individuals seeking addiction treatment.

**Methods:**

The cross sectional study was conducted on the sample recruited from three drug treatment centers in Pakistan. Face-to-face interviews were conducted with participants who met ICD-10 criteria for prescription drug dependence. Several aspects like substance use histories, negative health outcomes, patient attitude, pharmacy and physician practices also collected to predict the determinants of (PDD). Binomial logistic regression models examined the factors associated with PDD and PIDU.

**Results:**

Of the 537 treatment seeking individuals interviewed at baseline, close to one third (178, 33.3%) met criteria for dependence on prescription drugs. The majority of the participants were male (93.3%), average age of 31 years, having urban residence (67.4%). Among participants who met criteria for dependence on prescription drugs (71.9%), reported benzodiazepines as the most frequently used drug, followed by narcotic analgesics (56.8%), cannabis/marijuana (45.5%), and heroin (41.5%). The patients reported alprazolam, buprenorphine, nalbuphine, and pentazocin use as alternatives to illicit drugs. PDD was significantly negatively associated with injectable route (OR = 0.281, 95% CI, 0.079–0.993) and psychotic symptoms (OR = 0.315, 95% CI, 0.100, 0.986). This implies that PDD is less likely to be associated with an injectable route and psychotic symptoms in contrast to PIDU. Pain, depression and sleep disorder were primary reasons for PDD. PDD was associated with the attitude that prescription drugs are safer than illicit drugs (OR = 4.057, 95%CI, 1.254–13.122) and PDD was associated with being on professional terms (i.e., having an established relationship) with pharmaceutical drugs retailers for acquisition of prescription drugs.

**Discussion and conclusion:**

The study found benzodiazepine and opioid dependence in sub sample of addiction treatment seekers. The results have implications for drug policy and intervention strategies for preventing and treating drug use disorders.

## Introduction

For the few past decades, nonmedical use of prescription drugs has emerged as a global public health concern, particularly in high-income countries (1). According to the United Nations Office on Drugs and Crime (UNODC) World Drug Report, the non-medical use of prescription drugs is a major threat to public health and law enforcement worldwide (2). Nonmedical use of prescription drugs is a notable public health concern in United States (3), and other high income countries including Australia (4) and Canada (5).

Some pharmaceuticals, for example opioid pain relievers, share similar properties and have a similar potential for dependence and nonmedical use as heroin (6). Compulsive use and psychological dependence on some classes of pharmaceutical drugs cause serious consequences of health, and at times has accounted for more fatalities than cocaine and heroin combined in US (7). In North America, hydrocodone, oxycodone, codeine and tramadol are the main pharmaceutical opioids used for non-medical purposes. In Europe, fentanyl, methadone and buprenorphine reported to be misused (8). Whereas people of Africa and Middle East countries report mainly tramadol use (2). Globally, the variety of prescription drugs used, their use in different combinations, and their associated harms are rapidly changing, and this poses public health challenges, and challenges for clinical management (9) as research also found that medication adherence for psychiatric patients is a clinical concern (10).

The escalating global problem of dependence on prescription drugs offers challenges that vary from those of illicit drugs, and therefore needs innovative solutions (11, 12). There are an estimated 7.6 million people who use drugs in Pakistan with an estimated more than 800,000 who are thought to be drug dependent (13). Cannabis is the most common drug used in Pakistan, used by 4 million people. “Painkillers” (commonly opioids) are the next most common substance used by 1·69 million people (14).

A UNODC report stated that one in four people who use drugs in Pakistan report nonmedical use of prescription opioids (15). Other common pharmaceuticals used include pheniramine maleate, promethazine, diazepam, and temazepam (16). According to 2021 seizure data of Anti-Narcotics Force (ANF) Pakistan, 13,208 kg of opium, 2,219 kg of morphine, 4,360 kg of heroin, 43,423 kg of hashish, 0.05 kg of cannabis, 15 kg of cocaine, alprazolam 174 kg, and diazepam weighing 78 kg were seized (17).

Despite accumulating evidence of a rise in nonmedical use of prescription drugs globally, Pakistan has no reliable and routinely collected estimates of diversion of prescription drugs. United States, Canada, Australia and some European countries closely monitor and document the non-medical use of prescription drugs (18).

Since 2013 survey of Pakistan no national drug use survey has been conducted. Whereas the trends of drugs use have been changed and there is rapid rise in the use of synthetic drugs with illicit drugs. Therefore, scientific study is important to provide reliable data on current situation of drugs use, and a better understanding of prescription drug use problems in Pakistan needed to inform policy and clinical responses.

The aim of this study was to analyze the nonmedical use of prescription drugs used on their own or when used in combination with illicit drugs in Pakistan among treatment seeking people. Specifically, to:

1. Describe characteristics of treatment seeking people who meet criteria for prescription drug dependence
2. Identify which drugs are primary and secondary drugs of choice among people who meet criteria for prescription drug dependence.
3. Determine correlates of prescription drugs use, comparing those who use prescription drugs alone with those who report concomitant use of prescription and illicit drugs.

## Methods

### Study design

We conducted a multicenter cross-sectional study. The nonmedical prescription drug use is defined as any intentional use of a medication with intoxicating properties for reasons other than the purpose intended by the physician for a *bona fide* medical condition (19).

The term PDD adopted in this manuscript to refers to dependence on prescription drugs that is characterized by behavioral and other responses resulting in compulsions to take a drug, on a continuous or periodic basis in order to experience its psychological effects and at times to avoid the discomfort of its absence (20).

Likewise, the term prescription dependence and illicit drugs use (PIDU) demonstrates dependence on prescription drugs and illegal drugs use without differentiating the severity.

### Study settings

The current study includes the data from face to face interviews with treatment seeking people recruited in three drug treatment centers of Pakistan between September 2019 to March 2021; Nai Zindagi Hospital located in Multan city, Arrahma Hospital located in Multan city and Model addiction treatment and rehabilitation center (MATRC) located in Islamabad (capital city of Pakistan).

### Sampling, enrollment, and screening of study participants

Clinical staff in each center referred and recruited study participants, using a combination purposive sampling and chain-referral sampling. Eligibility for face to face interview was based on EMCDDA definitions and restricted to individuals who reported current (within the previous month), regular (more than or equal to 4 days per week) or intensive (daily) nonmedical use of a prescription drug and met ICD-10 criteria (21) for dependence on at least one prescription drug (22). Prescreening questionnaires for ICD-10 dependence were used to initially screen potential participants (23). Participants who appeared eligible at screening were invited to schedule an in-person visit at which eligible was confirmed and informed consent obtained. Those who reported use but not current, regular or intensive use according to EMCDDA definitions, were excluded from the analysis, to focus on the sample most likely to experience severe harm with prescription drugs.

**Figure 1:**
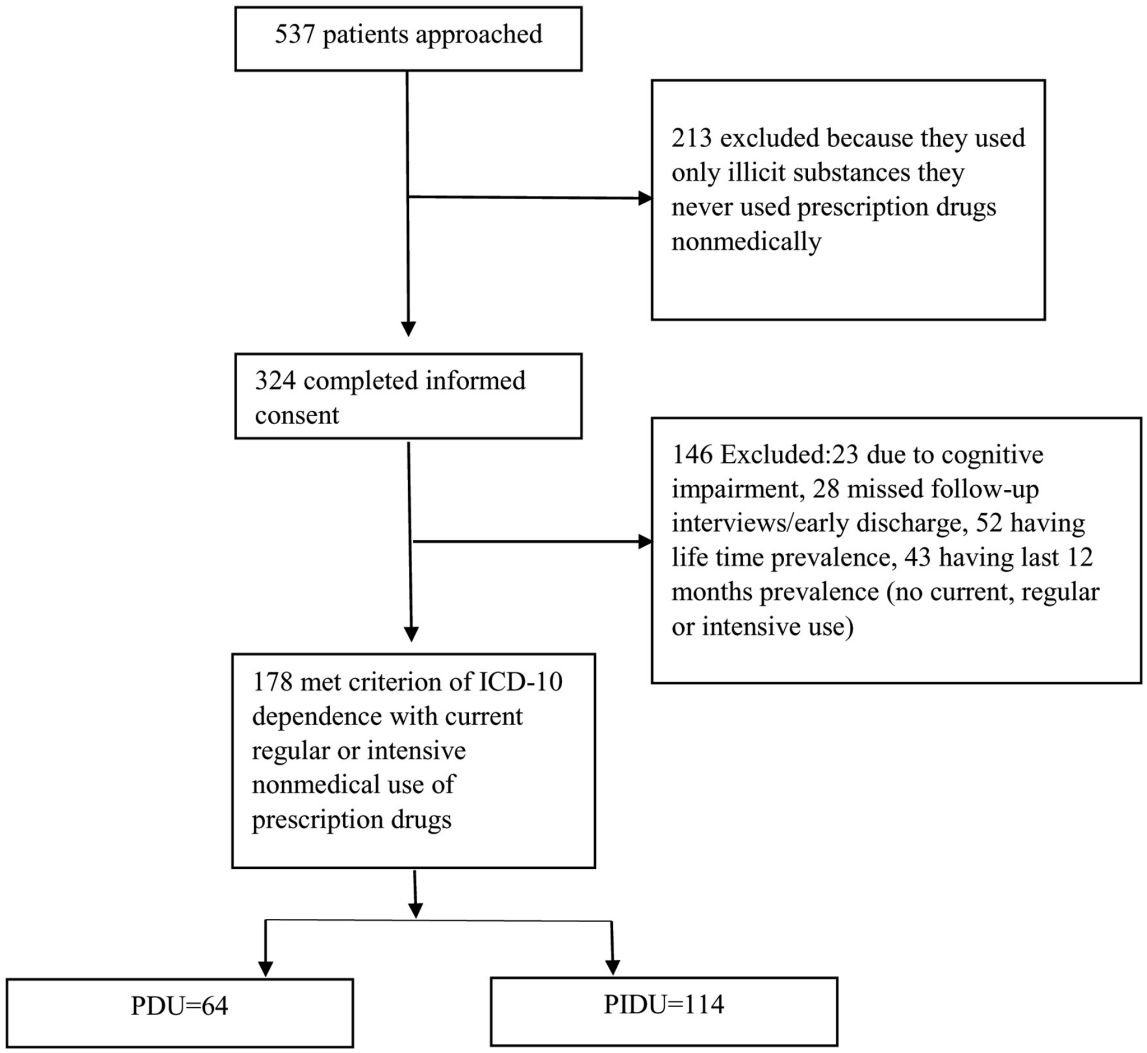
Sampling, enrollment, and screening of study participants.

In preliminary screening questions, 537 individuals seeking treatment were approached. Those who met criterion of ICD-10 dependence and reported current regular or intensive nonmedical use of at least one prescription drug were considered eligible. Other potential participants were excluded for reasons such as cognitive impairment preventing ability to given informed consent, missed follow-up interviews, just past 12 months and life time prevalence (meaning once in a year and once in life) pharmaceutical drug use, detail is shown in Figure 1.

### Measures

For preliminary screening, participants were asked if they had used various prescription drugs nonmedically and completed screening tool [Prescription drugs Use Questionnaire, Prescription Drugs Attitudes Questionnaire (PDAQ) validated instruments], and completed a checklist for dependence using ICD-10 criteria (21). We used a checklist for diagnosis of dependence based on ICD-10 criteria. To meet criteria for dependence according to the ICD-10, the following items were assessed each with a dichotomous response (Yes/No): a strong desire or sense of compulsion (strong desire), difficulties in controlling substance-taking behavior (difficulties in controlling), a physiological withdrawal state (withdrawal), evidence of tolerance (tolerance), need of larger doses to achieve the desired psychological effect, spending a disproportionate amount of time in obtaining, using, and recovering from drug use, continue to take the drug despite the problems (harmful consequences). The presence of three or more indicators was used to classify the patient as prescription drug dependent (23).

Identifying the extent and correlates of PDD was the primary focus of study. Several other aspects of drug use were also explored during interviews, including substance use histories, age of initiation, drug types used, frequency and duration of use, negative health outcomes, evaluation of pharmacy and physician practices associated with drug use. A series of structured questions captured patient characteristics including age of first use, patterns of nonmedical use, attitudes toward prescription drugs by using Prescription Drugs Attitudes Questionnaire (PDAQ) (24), reasons for use, and practices of acquiring prescription drugs through pharmacies and physicians.

### Patient characteristics

Patient characteristics including gender, age, marital status, residential level, education, occupation and monthly income collected from medical records, verified from the identification documents and confirmed during interviews. Survey data was deidentified after data collection was compete, in accordance with the Health Insurance Portability and Accountability Act of 1996 (HIPAA) Privacy Rule (25).

### Prescription drug use with primary and secondary choice of drugs

Participants were asked about use of any medication without a prescription to help symptoms such as pain, sleeping problems, improve mood, relieve stress, anxiety, or depression. Lifetime, annual prevalence, current use, regular use and daily use of prescriptions drugs documented using binary variables (yes/no).

The primary drug was defined as the “drug that causes the client most problems” and the secondary drugs are “the next most problematic drugs after that primary drug” (26). Participants were asked about number and types of drugs on which patients are currently, regularly or intensively dependent and each drug is identified as either their primary or secondary drug of choice and documented accordingly.

### Study size

We examined 31 predictors for modeling the PDD, the significance of these predictors tested with type 1 error (*α* = 0.05). The sample size of 167 was determined based on the estimated prevalence of prescription drug use among the addiction treatment-seeking population and the desired level of precision for the study results. The study aimed to achieve a precision level of ± 7.5% with a 95% confidence interval and power of the test to be 0.9.

### Statistical analysis

Data were analyzed using SPSS 20.0 statistical software data. Descriptive statistics used to describe continuous variables. The frequency and percentage calculated for categorical variables, mean and standard deviation for continuous variables that were normally distributed.

### Binomial logistic regression model

We performed a binomial logistic regression model, with PIDU as the reference category. The significant correlates and variables to be included in the model were guided by previous literature (27–29). Initial univariate analyses Chi-square test (for categorical variables) and *t*-tests (for continuous variables) were conducted and all variables, with those variables that were associated with PDD in the univariate analysis (*p* < 0.05) included in the multivariable binomial logistic regression model. We used a stepwise backwards elimination model where the results of the Wald test for individual parameters were examined for each variable. With each regression step, the least significant variables were removed from the model with only those variables associated with PDD, and PIDU (*p* < 0.05) kept in the final model.

### Ethical approval

The study adhered to the Declaration of Helsinki (30) and approved by the Institutional Review Boards (IRB) and Bio-Ethical Committee of Quaid-i-Azam University Islamabad with approval reference number PHM/2018/515 dated 28th February 2018. The data collection initiated after the official permission letters secured from administrators of each drug treatment center. The psychiatrists and psychologists explained the purpose and importance of the study to the voluntary participants before the interview sessions.

## Results

### Characteristics of current/regular/daily users of prescription drugs

Of the 537 treatment seeking individuals interviewed at baseline, close to one third (178, 33.3%) reported current, regular and intensive use and dependence on prescription drugs. Among this sub sample who met eligibility criterion (*n* = 178), 93.3% were male. The mean age was 31 years (*SD* = 9). About half (53.9%) of the respondents were married. Most (67.4%) lived in urban settlements and 64.0% lived in a major city. One in seven (14.6%) had no formal education. Most participants were unemployed with a median monthly income level was less than 30,000PKR (Pakistani rupee), IQR (InterQuartile Range) 29,250 PKR (equivalent to USD$186), which we defined as the cut off level for the low-income group. The most common age of first drug use was 10 to 15 years (reported by 23.0%), followed by 20–25 years of age (reported by 20.2%). Common routes of administration were oral 77.5%, injection 60.7%, smoked 44.4%, sniffed 33.1%, drink 30.3%, snort 10.7%, and inhaled 7.9%.

The study population was categorized into two mutually exclusive groups; participants having only prescription drugs dependence (PDD, *n* = 64, 36.0%) and those with dependence on prescription drugs and also reporting illicit drug use (PIDU) (*n* = 114, 64.0%). Table 1 represents the demographic characteristics and physical and mental health disorders for the two groups of participants, those with PDD and PIDU.

**Table 1:** Demographic characteristics and drug use disorders history of two groups of participants including individuals with prescription drug dependence (PDD) with and without concurrent illicit drug use.

Demographic characteristics and drugs use disorders		PIDU (REF) (*n* = 114)		PDD (*n* = 64)	
		Count	Column %	Count	Column %
Age	≤30 years	57	50.0%	23	35.9%
	≥30 years	57	50.0%	41	64.1%
Marital status	Married	68	59.6%	43	67.2%
	Single	46	40.4%	21	32.8%
Residential level	Outer regional/remote/rural	21	18.4%	6	9.4%
	major city/regional city	93	81.6%	58	90.6%
Education	Uneducated	53	46.5%	13	20.3%
	Educated	61	53.5%	51	79.7%
Occupation	Non-health professional	105	92.1%	42	65.6%
	Health professional	9	7.9%	22	34.4%
Monthly income categories	Low income group	52	45.6%	37	57.8%
	High income group	62	54.4%	27	42.2%
Drugs use pattern (oral)	No	19	16.7%	21	32.8%
	Yes	95	83.3%	43	67.2%
Drugs use pattern (injectable)	No	30	26.3%	23	35.9%
	Yes	84	73.7%	41	64.1%
GIT disorders	No	56	49.1%	14	21.9%
	Yes	58	50.9%	50	78.1%
Psychotic symptoms	No	35	30.7%	32	50.0%
	Yes	79	69.3%	32	50.0%
Nervous system disorders	No	76	66.7%	28	43.8%
	Yes	38	33.3%	36	56.3%
Cardiovascular system disorders	No	75	65.8%	52	81.3%
	Yes	39	34.2%	12	18.8%
Skin disorders	No	78	68.4%	37	57.8%
	Yes	36	31.6%	27	42.2%
Urogenital disorders	No	68	59.6%	44	68.8%
	Yes	46	40.4%	20	31.3%
Psychological disorders (physical)	No	14	12.3%	10	15.6%
	Yes	100	87.7%	54	84.4%
Psychological disorders (emotional)	No	16	14.0%	10	15.6%
	Yes	98	86.0%	54	84.4%
Psychological disorders (behavioral)	No	23	20.2%	16	25.0%
	Yes	91	79.8%	48	75.0%

### Primary and secondary choice of drugs and specific prescription drugs misused reported by drugs users

**Figure 2:**
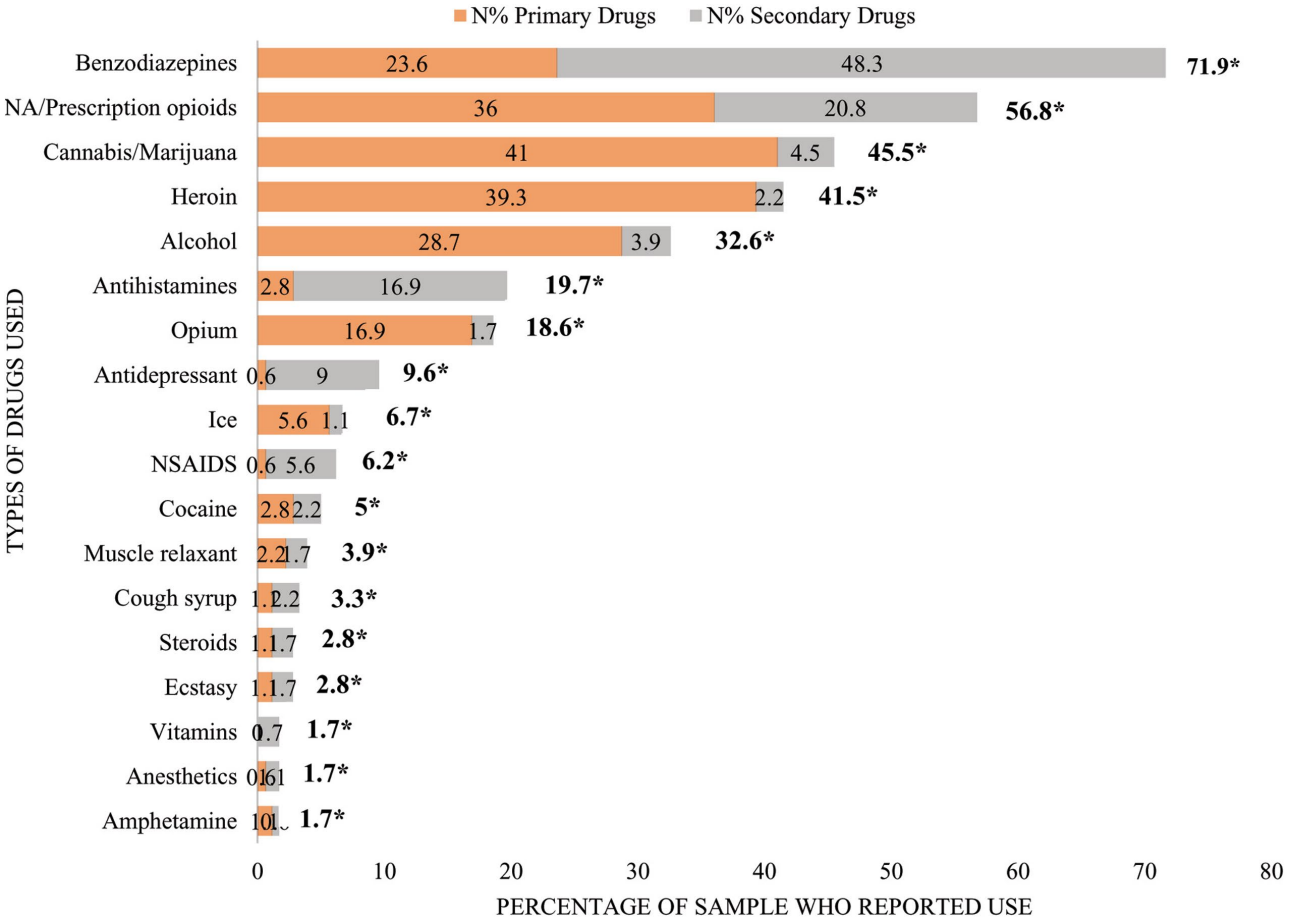
Primary and secondary choice of drugs reported by patients with current non-medical use. *Represents total percentage of each drug used as either primary choice of drugs or secondary choice. NA/prescription opioids means narcotics analgesics/prescription opioids.

The primary and secondary choice of drug (including both prescription or illicit drugs) by clients are shown in Figure 2. The maximum number of primary and secondary drugs used by respondents was 8, and the mean number of drugs used was 3. The commonly used drugs during last 30 days often called “current use,” reported regular use (an average of ≥4 days per week, and “daily use” an indicator of intensive use) by the study participants are benzodiazepines, prescription opioids/narcotics analgesic and cannabis/marijuana.

Benzodiazepines were the most common prescription drug used, reported by 71.9% of participants. One in four participants (23.6%) used benzodiazepines as their primary drug and half (48.3%) selected it as secondary drug. Over half (56.8%) of the study sample reported use opioid analgesics/narcotics analgesics. Of these, one in three (36.0%) reported opioid analgesics/narcotics analgesics as their primary drug while 20.8% reported opioid analgesics as their secondary drug.

The majority of the study population reported non-medical use of prescription drugs specifically including: alprazolam, diazepam, bromazepam, clonazepam, nalbuphine, buprenorphine and pentazocin either alone or in combination with illicit drugs. Other pharmaceuticals misused reported were temazepam, lorazepam, midazolam, lormetazepam, zolpidem tartarate, tramadol, oxycodone, amitriptyline, escitalopram, fluoxetine, and paroxetine (Table 2).

**Table 2:** Commonly used pharmaceuticals by addiction treatment seeking individuals.

Pharmaceutical drug class	Common used drugs	Percentage (n%)
Benzodiazepines	Alprazolam Diazepam Bromazepam Clonazepam Temazepam Lorazepam Midazolam Lormetazepam Zolpidem tartarate	25.8 20.2 15.2 9.6 7.9 5.6 2.2 1.7 0.6
Opioids/Narcotics analgesics	Nalbuphine Buprenorphine Pentazocin Tramadol Oxycodone	21.9 16.9 5.6 4.50 1.1
Antidepressants	Amitriptyline Escitalopram Fluoxetine Paroxetine	1.7 1.1 0.6 0.6

### Binary logistic regression model for prescription drugs users compared to those using prescription and illicit drugs

Bivariate results for associated factors of PDD are listed in Table 3. High monthly income (OR = 0.067) and injection mode of use (OR = 0.095) were significantly negatively correlated with PDD. Whereas presence of self-reported or diagnosed skin disorders (OR = 14.412) and drug related psychological problems mainly emotional problems (OR = 22.229) were more likely associated with PDD. The study revealed the primary reasons for PDD were depression (OR = 6.455) and sleep disorders (OR = 20.031). PDD patients more likely to have established relationships with drugs retailers (OR = 0.035) and to report prescription drug initiation by physician, prescribed for anxiety or depression (OR = 6.377).

**Table 3:** Bivariate analysis of the possible factors in association with PDD and PIDU.

Factors	*B*	S.E.	Wald	*p*-value	Odds ratio	95% odds ratio CI	
						Lower	Upper
Age	−0.687	1.286	0.285	0.593	0.503	0.04	6.259
Marital status	−1.966	1.266	2.414	0.12	0.14	0.012	1.673
Residential level	2.367	1.359	3.035	0.082	10.67	0.744	153.115
Education	0.559	1.083	0.266	0.606	1.748	0.209	14.597
Occupation	0.438	1.261	0.121	0.728	1.55	0.131	18.352
Monthly income	−2.707	1.024	6.985	0.008	0.067	0.009	0.497
Oral	1.132	1.317	0.739	0.39	3.102	0.235	41.005
Injection	−2.35	1.103	4.544	0.033	0.095	0.011	0.828
GIT disorders	−0.006	1.17	0	0.996	0.994	0.1	9.841
Psychotic symptoms	−1.642	1.293	1.611	0.204	0.194	0.015	2.444
Nervous system disorders	1.834	1.128	2.647	0.104	6.261	0.687	57.064
Cardiovascular system disorders	0.385	1.313	0.086	0.77	1.469	0.112	19.264
Skin disorders	2.668	1.354	3.885	0.049	14.412	1.015	204.594
Urogenital disorders	−1.103	1.234	0.799	0.371	0.332	0.03	3.726
Drug related psychological problems DRPP (physical)	−0.298	1.274	0.055	0.815	0.742	0.061	9.011
Drug related psychological problems DRPP (emotional)	3.101	1.51	4.219	0.04	22.229	1.153	428.641
Drug related psychological problems DRPP (behavioral)	−2.103	1.411	2.221	0.136	0.122	0.008	1.94
Primary reason: pain	0.84	1.055	0.635	0.426	2.317	0.293	18.325
Primary reason: depression	1.865	0.896	4.332	0.037	6.455	1.115	37.371
Primary reason: sleep disorder	2.997	1.394	4.624	0.032	20.031	1.304	307.697
Patient attitude: fewer side effect than street drugs	−0.689	1.074	0.412	0.521	0.502	0.061	4.116
Patient attitude: easy to get due to ease of availability	−0.055	1.046	0.003	0.958	0.946	0.122	7.357
Patient attitude: safer use than illegal drug	1.377	1.01	1.857	0.173	3.962	0.547	28.711
Patient attitude: can be used as study aid	1.393	1.324	1.107	0.293	4.025	0.301	53.902
Prescription verification while dispensing	−0.367	1.648	0.05	0.824	0.692	0.027	17.5
Price of prescription drugs	−1.131	1.317	0.738	0.39	0.323	0.024	4.263
Monetary compensation to drugs retailers	0.787	1.387	0.322	0.57	2.198	0.145	33.315
Professional terms with drugs retailers	2.226	1.053	4.464	0.035	9.259	1.175	72.99
Prescribed by physician for muscle pain or headache	0.787	1.193	0.436	0.509	2.198	0.212	22.761
Prescribed by physician for anxiety or depression	1.853	1.095	2.861	0.091	6.377	0.745	54.578
Constant	−6.433	3.28	3.846	0.05	0.002		

Binary logistic model identified predictors of PDD (Table 4). The multivariable binary logistic model applied on the study population was categorized into two mutually exclusive groups; participants having only prescription drugs dependence (PDD, *n* = 64, 36.0%) and those who used/dependence on prescription drugs and illicit drugs (PIDU) (*n* = 114, 64.0%). The results of the multivariable binary logistic model revealed that those classified as PDD were less likely to report an injected route of administration, or psychotic symptoms compared with PIDU. PDD were more likely to report pain, depression and sleep disorder as the primary reasons for use of prescription drugs. Participants classified as PDD were more likely to perceive that prescription drugs were safer to use than illegal drugs. Among the variables examining prescription drugs acquisition, PDD were more likely to report being on professional terms and established relationships with pharmaceutical drugs retailers.

**Table 4:** Influential factors in association with prescription drugs use using binary logistic regression model.

Variables	PDD (*n* = 64)	PIDU (REF) (*n* = 114)	Odds ratio	95%CI	Adjusted odds ratio	95%CI
Injectable route of administration (Ref: No)	(*n* = 41) 64.1%	(*n* = 84) 73.7%	0.637	0.329, 1.231	0.281	0.079, 0.993
GIT disorders (Ref: No)	(*n* = 50) 78.1%	(*n* = 58) 50.9%	3.448	1.717, 6.924	2.685	0.816, 8.841
Psychotic symptoms (Ref: No)	(*n* = 32) 50.0%	(*n* = 79) 69.3%	0.443	0.443, 0.833	0.315	0.100, 0.986
Nervous system disorders (Ref: No)	(*n* = 36) 56.2%	(*n* = 38) 33.3%	2.571	2.571, 4.823	3.187	0.987, 10.296
Primary reason for use: pain (Ref: No)	(*n* = 19) 29.7%	(*n* = 18) 15.8%	2.252	2.252, 4.699	4.443	1.236, 15.969
Primary reason for use: depression (Ref: No)	(*n* = 42) 65.6%	(*n* = 25) 21.9%	6.796	6.796, 13.420	4.449	1.493, 13.252
Primary reason for use: sleep aid (Ref: No)	(*n* = 46) 71.9%	(*n* = 57) 50.0%	2.556	1.325, 4.930	5.086	1.500, 17.248
Patient attitude: safer to use than illegal drugs (Ref: Disagree)	(*n* = 43) 67.2%	(*n* = 42) 36.8%	3.510	1.840, 6.696	4.057	1.254, 13.122
Evaluation of pharmacy practice: professional terms with retailers (Ref: No)	(*n* = 34) 54.0%	(*n* = 51) 45.9%	1.379	0.742, 2.565	4.638	1.373, 15.660

## Discussion

To our knowledge, this is the first detailed study examining dependence on prescription drugs in Pakistan among a treatment seeking sample. Almost one in three people screened reported current and regular or intensive prescription drug use and met criteria for prescription drug dependence. Our study revealed 33.3% of prevalence of non-medical use of prescription drugs in contrast to the results of study conducted in Uganda reporting prevalence of 11.4% patients with history of controlled prescription drugs non-medical use (31).

Findings demonstrate high rates of dependent benzodiazepine use, reported by almost three quarters of the sample followed by dependent use of prescription opioids, reported by more than half of the sample. Similar to our findings benzodiazepines found to be the most prevalent prescription drugs used by drugs treatment seeking population of Singapore (32). The common use of and dependence to benzodiazepines may have implications for their regulation and supply in Pakistan.

Those reporting nonmedical use of prescription medicines report use for pain, sleep and depression, which may indicate that these comorbidities are common among this group. In another study conducted in Pakistan, sleep induction and alleviation of stress reported by medical students as motives of using drugs (33). Given this, future studies may explore the capacity of treatment services to address pain and mental illness among people experiencing prescription drug dependence.

Our study identified some parallels to research conducted already in high-income countries. An international review found that benzodiazepines were commonly used as secondary drug among people seeking treatment for substance use disorders (34). An European study found that 12% of entrants for opioid-related problems reported benzodiazepines as a secondary problem drug (35). Among those in opioid substitution therapy, the prevalence of benzodiazepine use ranged from approximately 40% in the United States (36), to 70% in Germany (37). In the Asia-pacific region there is paucity of accurate data due to lack of national surveys, yet misuse of prescription opioids and benzodiazepines is reported (38). Nawaz et al. found that half of the medical students (49.3%) reported benzodiazepine use for psychological stress (39).

Although benzodiazepines and opioids analgesics are controlled prescription drugs in Pakistan, possible explanations for their common misuse in this study may be the illegal selling by retailers without a prescription (40), or through acquisition via multiple prescribers is also common, due to a lack of online medical records (41). Alprazolam was the most common benzodiazepine reported in this sample. This is of particular concern given the greater propensity for alprazolam to cause dependence and overdose harm (42).

Given the common contribution of benzodiazepines to fatal overdoses (43), our findings may suggest that greater controls over benzodiazepine availability are warranted. For example, regulatory changes to restrict alprazolam access in Australia have led to decline in alprazolam use among people who inject drugs (44).

We found participants more frequently reported use of prescription opioids analgesics as their primary drug of choice compared to benzodiazepines. This is in contrast to India where only 40.6% of substance use is accounted for opioids, with (12.1%) being pharmaceutical opioids (38). Use of prescription opioids such as nalbuphine, buprenorphine, and pentazocine were common in Pakistan, contrasting to the more common use of hydrocodone and oxycodone use in the USA (45). The common use of atypical opioids in Pakistan may make it challenging to translate findings from US treatment research to settings such as in Pakistan where the most commonly used opioids are atypical opioids such as nalbuphine.

We found common drivers of PDD were pain, depression and sleep disorders, which is consistent with previous studies that reported stress, anxiety, and sleep among the motives of using opioids (46). This is also similar to US reported pain relief was the most common reason for misusing prescription opioids (47). Similarly, major risk factors for harms related to opioid misuse reported by Lalic et al., were chronic non cancer pain, substance abuse, mental disorders, and concomitant use of centrally acting substances, benzodiazepines and antidepressants (48). Being custodian of legitimate supply of medicines there is need of training the pharmacists and public health professionals in new practices for clinical management of drug dependencies.

Perhaps unsurprisingly, we found use of drugs by injection was more common among PIDU, confirming that delivering harm reduction interventions is a priority for people who are dependent on prescription drugs alongside illicit drugs. Previous research conducted in Los Angles and New York has also shown the link between injection of opioids and prescription opioid use (49). Guarino et al., showed that people who use prescription opioids switched to heroin by injections (64%) after initial prescription opioid use (50).

We found adolescent age (10–15 years) was critical risk period for the initiation of drug use. Several international studies identify greater risk of developing dependence to substances like cannabis, heroin, psychostimulants and cocaine for individuals who initiate drug use in the early years of adolescence (51–53). The US National Survey on Drug Use and Health data indicate that the adults whose incidence age of cannabis use was 14 years or younger has 6 times higher percentage of drugs dependence than the adults having incidence age of 18 years or older (54).

Prevention of nonmedical use of and dependence to prescription drugs, and related harms remains a global challenge, particularly given the need to reduce diversion while guaranteeing the accessibility of medicines for therapeutic use (55). This challenge appears greater for countries like Pakistan where strict international drug controls restrict the access to many medicines such as morphine for humane pain relief, with 80% population having insufficient access to morphine for severe pain (56).

The recommendations for specific measures and harm reduction interventions that can be implemented to strengthen the regulation of prescription drugs in Pakistan and reduce their misuse are given below:

- The study reveals the illicit drugs users approach pharmacies for acquiring prescription drugs for the symptomatic relief of sleep disturbance, depression and stress symptoms. This leads to illicit sale practices of controlled prescription drugs. Pharmacists can serve as the first line of defense in recognizing patterns of prescription drug dependence and supporting access to appropriate treatment. It is strongly recommended that the pharmacists at retail outlets should be trained to identify problematic drugs use and assist people to access evidence based harm reduction services including needle and syringe program, opioid agonist treatment, take-home naloxone program and refer them to drug detoxification centers.
- Sale of Controlled Drugs/Schedule G Drugs at Community Pharmacies of Pakistan should be strictly regulated by appropriate authorities.
- Introduction of Centralized prescription network (CPN) to detect therapeutic duplication of drugs and Prescription drug monitoring programs (PDMPs), as a state-run electronic databases used to track the prescribing and dispensing of controlled prescription drugs to patients, are also important tools for preventing and identifying prescription drug misuse.
- Recognizing that adolescents aged 10–15 years are a high risk group for initiating drug use, they should be encouraged to have access to evidence-based harm reduction services and promote life styles that support healthy brain and personality development. Youth should be engaged in the activities that is helpful in personal growth rather than only satisfaction of need arousal (57). Further School-based prevention and skill-training interventions are effective tools to reduce substance use among adolescents.

### Limitations of this study

A strength of study is the recruitment from multiple treatment sites, but as the study represents only treatment-seeking individuals, the results cannot be generalized to other populations of people who use prescription drugs outside this setting. Common limitations with self-reported, such as social desirability bias may exist. Finally, our study focused on extensive but finite range of variables. There may be other factors that differentiate those who use prescription drugs alone and those that used only illicit drugs that were not measured.

## Conclusion

This study highlighted the common dependence to prescription drugs including benzodiazepines and prescription opioids/narcotic analgesics in combination with illicit drug use. Nearly one third of the total population of treatment seeking individuals approached met criteria for dependence on prescription drugs. Developing strategies to reduce the nonmedical use of prescription drugs and their related harms without limiting access for all essential medicines remains a challenge.

## Data availability statement

The original contributions presented in the study are included in the article/Supplementary material, further inquiries can be directed to the corresponding authors.

## Ethics statement

The studies involving human participants were reviewed and approved by the Institutional Review Boards (IRB) and Bio-Ethical Committee of Quaid-i-Azam University Islamabad. Written informed consent to participate in this study was provided by the participants’ legal guardian/next of kin.

## Author contributions

AN and AK conceptualized the study and designed the methodology. TM as biostatistician assisted in formal statistical analysis. AN wrote the first draft of the manuscript. AAb reviewed and edited the manuscript. SN provided input on the analysis and interpretation of the results, and assisted with revising drafts of the manuscript in collaboration with AN. AK supervised the progress of the study. AN, AAh, and WU contributed in revisions suggested by the reviewers, and preparing final version of the manuscript. All authors approved the final copy.

## Conflict of interest

The authors declare that the research was conducted in the absence of any commercial or financial relationships that could be construed as a potential conflict of interest.

## Publisher’s note

All claims expressed in this article are solely those of the authors and do not necessarily represent those of their affiliated organizations, or those of the publisher, the editors and the reviewers. Any product that may be evaluated in this article, or claim that may be made by its manufacturer, is not guaranteed or endorsed by the publisher.
